# Performance and clinical implications of non-invasive prenatal testing for rare chromosomal abnormalities: a retrospective study of 94,125 cases

**DOI:** 10.3389/fmolb.2025.1645223

**Published:** 2025-08-20

**Authors:** Haimei Qi, Haijun Chen, Zhuling Zhang, Jinhui Gan, Huifeng Liu, Xianping Yuan, Fuyu Luo, Junkun Chen, Shichun Shen

**Affiliations:** ^1^ Clinical Laboratory, Ganzhou Maternal and Child Health Hospital, Ganzhou, China; ^2^ Department of Medical Genetics, Ganzhou Maternal and Child Health Hospital, Ganzhou, China; ^3^ Obstetrical Department, Ganzhou Maternal and Child Health Hospital, Ganzhou, China

**Keywords:** noninvasive prenatal testing, rare chromosomal abnormalities, positive predictive value, true fetal mosaicism, pregnancy outcomes, small-for-gestational-age

## Abstract

**Background:**

Non-invasive prenatal testing (NIPT) has demonstrated robust performance in detecting common trisomies and copy number variations. However, its clinical utility for rare chromosomal abnormalities (RCAs) remains controversial due to low positive predictive value (PPV).

**Methods:**

This study retrospectively analyzed the data of 94,125 cases that underwent NIPT at Ganzhou Maternal and Child Health Hospital in China. This dataset was used to evaluate NIPT performance in RCAs detection and track pregnancy outcomes of positive cases.

**Results:**

In the cohort of 94,125 pregnancies undergoing NIPT, 336 cases (0.36%) were found to carry RCAs. Among them, 102 cases underwent validation through karyotyping and/or chromosome microarray analysis. Of the 102 validated cases, seven were true positives (PPV = 6.86%). Additionally, 3 cases exhibited uniparental disomy consistent with the NIPT-reported chromosomal anomalies. Of 268 singleton neonates, 68 (25.37%) were small-for-gestational-age.

**Conclusion:**

This study found that most NIPT-detected RCAs were associated with maternal age, while Trisomy seven occurred independently of maternal age. Concurrent use of karyotyping and chromosome microarray analysis, rather than karyotyping alone, mitigates culture-related bias and enhances the PPV. Both biological and methodological factors contribute to the low PPV of NIPT for RCAs. Despite a low PPV, pregnancies with NIPT-indicated RCAs showed elevated risks of fetal loss, small-for-gestational-age, and uniparental disomy, though not preterm birth. Thus, NIPT-detected RCAs retain clinical significance for risk stratification and pregnancy management.

## 1 Introduction

Chromosomal aneuploidy, defined as a condition in which the number of one or several chromosomes in a cell deviates from the normal diploid state (Li and Zhu, 2022), primarily arises from chromosomal nondisjunction during meiosis or mitosis. This genetic abnormality is a leading cause of birth defects, spontaneous miscarriages, and genetic disorders (Klaasen and Kops, 2022). Clinical chromosomal aneuploidies include trisomy syndromes such as Down syndrome (trisomy 21), Edwards syndrome (trisomy 18), and Patau syndrome (trisomy 13) (Antonarakis et al., 2020; Outtaleb et al., 2020; Schlosser et al., 2023), as well as sex chromosome aneuploidies (SCAs) like Turner syndrome (45,X), Klinefelter syndrome (47,XXY), Triple X syndrome (47,XXX), and XYY syndrome (Berglund, Stochholm and Gravholt, 2020). In contrast, most other autosomal aneuploidies frequently cause early spontaneous miscarriages and are rarely observed in live births. These rare chromosomal abnormalities (RCAs) are categorized as Rare Autosomal Monosomies (RAMs) and Rare Autosomal Trisomies (RATs) (M. Gug et al., 2024; Lannoo et al., 2022).

In recent years, non-invasive prenatal testing (NIPT), which analyzes cell-free fetal DNA (cffDNA) in maternal circulation via next-generation sequencing, has been widely implemented for detecting fetal chromosomal abnormalities (Dungan et al., 2023). Beyond its established diagnostic accuracy for trisomy 21, 18, and 13, accumulating evidence supports NIPT application for SCAs and copy number variations (CNVs) (Holesova et al., 2024; Johnston et al., 2023; Kim et al., 2024; Wang et al., 2024; Ye et al., 2021). However, the efficacy of NIPT in detecting RCAs remains controversial (Dungan et al., 2023). Many studies have reported extremely low positive predictive values (PPV) for NIPT in identifying RCAs (Lin et al., 2022; Van Den Bogaert et al., 2021), while others have found associations between NIPT-indicated RCAs and adverse pregnancy outcomes (Xiang et al., 2023; Yan et al., 2025). These controversies highlight the need for further clinical data to comprehensively evaluate the clinical utility and limitations of NIPT in RCAs screening. Large-scale studies provide valuable evidence to guide optimal management strategies for such cases (C. Gug et al., 2022), thereby improving evidence-based clinical decision-making for complex prenatal findings.

In this study, we conducted a retrospective analysis of 94,125 NIPT results to evaluate the performance of NIPT in screening for RCAs, investigate pregnancy outcomes of positive cases and explore the molecular mechanisms underlying false-positive results. This study aims to provide clinical evidence for the application of NIPT in RCAs screening.

## 2 Materials and methods

### 2.1 Participant recruitment

This study was conducted as a single-center retrospective analysis at Ganzhou Maternal and Child Health Hospital in China. A total of 94,125 pregnant women who underwent NIPT at the hospital between April 2015 and December 2024 were enrolled. The study protocol was reviewed and approved by the Ethics Committee of Ganzhou Maternal and Child Health Hospital (Approval Number: 2024099). All participants were informed about the testing methodology, conditions covered by the screening, limitations, and associated risks. Written informed consent was obtained from each participant, and all procedures were performed in accordance with relevant guidelines and regulations.

### 2.2 NIPT

Peripheral venous blood samples were collected from each pregnant woman using two protocols: 5 mL in EDTA-anticoagulated tubes or 5–10 mL in cell-free nucleic acid preservation tubes. Plasma was separated via two-step centrifugation with an Eppendorf 5810R centrifuge (Eppendorf, Germany): first at 1,600 × g (4° C), then at 16,000 × g (4° C). cffDNA was extracted from plasma using the QIAamp DSP DNA Blood Mini Kit (Qiagen). Extracted DNA underwent end repair using the Ion Plus Fragment Library Kit (Life Technologies), followed by magnetic bead-based size selection (<230 bp) to enrich cffDNA fragments. Size-selected DNA was then ligated to adapters and amplified via PCR. Library quantification was performed using quantitative PCR, with concentrations normalized through dilution to ensure uniform sequencing density. Pooled libraries were sequenced on the Bioelectron-seq 4000 platform (CapitalBio, China; CFDA registration NO. 20153400309). Following sequencing, the data were analyzed using NIPT Data Analysis Management Software (CapitalBio Genomics, China) to generate chromosomal Z-scores for each sample, with the core algorithm employing a generalized function for Z-score calculation. Raw data were filtered according to the following criteria: read mean length >100 bp, sequencing quality score (Q20) >50%, GC content between 38% and 45%, and cffDNA fraction ≥4%. Clean reads were aligned to the human reference genome (hg19). Following alignment, low-quality alignments and PCR duplicates were removed to obtain unique reads; each sample yielded a minimum of 3.5 million unique reads. Chromosomes were segmented into 20-kb bins. The percentage of unique reads relative to autosomal reads was calculated, with RCAs determined by |Z-score| >3 thresholds based on statistical deviation analysis. Further methodological details are described in our prior publication (Shen et al., 2024).

### 2.3 Prenatal diagnosis

All positive cases were provided with genetic counseling. Following informed consent, amniocentesis was performed at 16–22 weeks of gestation to obtain 20–30 mL of amniotic fluid. The amniotic fluid samples were cultured for chromosomal karyotyping using G-banding at a 400-band resolution or directly analyzed by chromosome microarray analysis (CMA) using CytoScanTM 750K (Affymetrix, United States).

### 2.4 Copy number variations sequencing

We collected placental tissues from pregnant women with false-positive NIPT results confirmed by prenatal diagnosis and performed copy number variations sequencing (CNV-seq). Placental tissues were obtained after delivery or induced abortion with informed consent from the participants. Genomic DNA was extracted using the QIAamp DSP DNA Blood Mini Kit (Qiagen). Extracted DNA underwent PCR amplification and library preparation. The sequencing libraries were subjected to circularization reactions and DNA nanoball preparation. Sequencing was conducted on the MGISEQ-2000 platform (MGI, Shenzhen, China). Raw sequencing data were aligned to the human reference genome (hg19) and analyzed using the Halos software to identify chromosomal abnormalities.

### 2.5 Pregnancy follow-up

All pregnant women were followed up via telephone or electronic medical record systems. For cases with RCAs confirmed by prenatal diagnostic amniotic fluid karyotyping and CMA, pregnancy outcomes and neonatal chromosomal karyotyping were recorded. Additionally, pregnancy complications and outcomes were monitored in two distinct cohorts: (1) those with false-positive RCAs results from prenatal testing, and (2) NIPT-positive cases who declined further invasive diagnostic procedures. The primary observational endpoints included birth weight, gestational age at delivery, and clinically significant developmental anomalies. According to the *Growth Assessment Standards for Newborns at Different Gestational Ages* issued by the National Health Commission of China in 2022 (National Health Commission of the People’s Republic of China, 2022), neonates were categorized as small-for-gestational-age (SGA) if their birth weight fell below the 10th percentile for their gestational age, and as preterm birth if delivered prior to 37 weeks of gestation.

### 2.6 Statistical analysis

Statistical analyses were performed using SPSS version 27.0 (IBM Corp., Armonk, NY, United States). Continuous variables are presented as mean ± standard deviation and compared using Student’s t-test; categorical variables are expressed as percentages (%) and analyzed using the chi-square test. Statistical significance was defined as P < 0.05.

## 3 Results

### 3.1 Positive cases and classification

**FIGURE 1:**
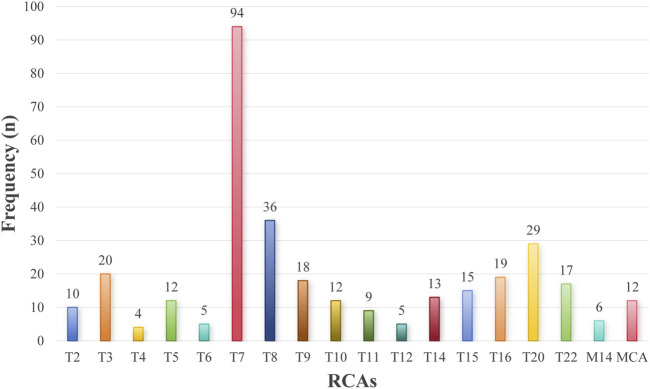
Frequency of RCAs detected by NIPT. TX, Trisomy X (X = 2, 3, 4 … 20, 22). M14, Monosomy 14. MCA, Multiple chromosomal abnormalities.

Among 94,125 NIPT samples, 336 RCAs-positive cases were detected (positive rate: 0.36%), comprising 330 RATs (98.21%) and 6 RAMs (1.79%; all involving monosomy 14). RATs predominantly affected single chromosomes (318 cases, 96.36%), with only 12 cases (3.64%) showing multiple abnormalities. Trisomy 7 (T7) was the most prevalent RCAs (N = 94, 27.98%), followed by trisomy 8 (N = 36), trisomy 20 (N = 29), and trisomy 3 (N = 20) (Figure 1). Notably, no abnormalities of chromosomes 1, 17, or 19 were observed (Supplementary Table S1).

### 3.2 Clinical characteristics and analysis of positive cases

Among the 336 RCAs-positive cases, 330 cases were singleton pregnancies and six were twin pregnancies, with gestational ages ranging from 12^+0^ to 32^+5^ weeks and maternal ages spanning 19–46 years. The indications for NIPT included advanced maternal age (≥35 years at expected delivery date) in 34.23% (115/336) of cases, high-risk or borderline-risk serum screening in 36.90% (124/336), ultrasound abnormalities in 3.57% (12/336) and patient-requested testing without clinical indications in 30.65% (103/336). The mean maternal age of RCAs-positive cases was 31.55 ± 4.95 years. Subgroup analysis revealed that T7 cases (N = 94) had a younger mean age of 29.72 ± 5.34 years, while other RCAs (N = 242) showed a mean age of 32.26 ± 5.84 years (Table 1).

**TABLE 1:** Comparative analysis of maternal age across distinct NIPT result categories.

NIPT result	Frequency	Percent	Maternal age (years)		
			Mean ± SD	Minimum	Maximum
RCAs	336	0.36%	31.55 ± 4.95	19	46
T7	94	0.10%	29.72 ± 5.34	19	41
non-T7 RCAs	242	0.26%	32.26 ± 5.84	19	46
Common trisomies	481	0.51%	32.47 ± 6.46	17	47
SCAs	344	0.37%	30.88 ± 5.97	17	45
CNVs	361	0.38%	29.21 ± 5.74	16	46
Negative	92,603	98.38%	30.42 ± 5.62	14	54

**FIGURE 2:**
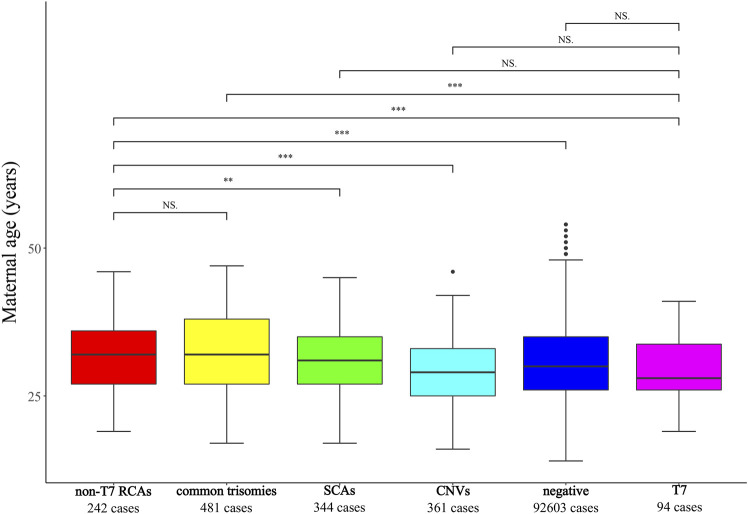
Comparative maternal age analysis. Non-T7 RCAs: other rare chromosomal abnormalities except trisomy 7. SCAs, sex chromosome aneuploidies; CNVs, copy number variations.***indicates P < 0.001. **indicates P < 0.01. ^NS^indicates P > 0.05.

Maternal age comparison showed that T7 cases showed no significant difference from SCAs-positive (P = 0.09), CNVs-positive (P = 0.44), or NIPT-negative cohorts (P = 0.23). However, T7 cases were significantly younger than common trisomy cases and non-T7 RCAs (both P < 0.05). Conversely, non-T7 RCAs exhibited no significant age difference compared to common trisomies (P = 0.67; Figure 2) but were significantly older than T7 cases, SCAs-positive, CNVs-positive, and NIPT-negative groups (all P < 0.05). Reference groups included: common trisomies (T21/T18/T13, n = 481; 32.47 ± 6.46 years), SCAs-positive (n = 344; 30.88 ± 5.97 years), CNVs-positive (n = 361; 29.21 ± 5.74 years), and NIPT-negative cases (n = 92,603; 30.42 ± 5.62 years).

### 3.3 Prenatal diagnostic outcomes

Among 336 RCAs-positive cases, 102 pregnant individuals underwent prenatal diagnosis, resulting in a prenatal diagnostic rate of 30.36% (102/336). Seven cases were confirmed as true fetal mosaicism for RATs (Table 2), resulting in a PPV of 6.86% (7/102). Notably, three cases initially showed no RATs in standard karyotyping (30 metaphases analyzed). After CMA revealed mosaicism, extended metaphase counting identified RATs mosaicism in two cases at lower proportions than CMA estimates (Supplementary Figure S1), while one case remained undetected (Supplementary Figure S2). CMA also detected three cases of uniparental disomy (UPD), with UPD chromosomes concordant with NIPT-reported RATs. Additionally, four discordant cases were identified: one NIPT-reported T14 case was diagnosed as 46,XX,−14,+rob(14; 21)(T21); one NIPT-reported trisomy 15 case revealed 45,XN,der(14; 15)(q10; q10)[109]/46,XN[8] (Robertsonian translocation); one NIPT-reported trisomy 20 case showed 46,XN,inv(20)(p13q13.1) (pericentric inversion); and one NIPT-reported trisomy 15 case was diagnosed as 47,XXY[21]/46,XY[69] (SCAs mosaicism).

**TABLE 2:** NIPT results, prenatal diagnosis outcomes, and pregnancy outcomes in true fetal mosaicism, uniparental disomy, and discordant cases.

Case ID	NIPT	Prenatal diagnosis		Pregnancy outcome			
		Karyotyping	CMA	Gestational age (weeks)	Sex	Weight (g)	Growth assessment
Group: true fetal mosaicism							
82	T9	47,XN,+9[7]/46,XN[67]	Not available	37^+6^	Male	2,170	SGA
134	T2	47,XN,+2[1]/46,XN[60]	58% T2 mosaicism	Termination of pregnancy			
204	T15	NA	39% T15 mosaicism	35^+2^	Female	1,900	SGA and PTB
235	T9	47,XN,+9[10]/46,XN[67]	15% T9 mosaicism	Termination of pregnancy			
244	T22	NA	23% T22 mosaicism	Termination of pregnancy			
329	T16	47,XN,+16[4]/46,XN[81]	21% T16 mosaicism	Termination of pregnancy			
333	T16	46,XN[130]	10% T16 mosaicism	30^+0^	Female	980	SGA and PTB
Group: uniparental disomy							
80	T22	46,XN	arr[hg19] chr22 × 2 hmz	33^+0^	Female	1,000	SGA and PTB
140	T9	46,XN	arr[hg19] chr9x2 hmz	Termination of pregnancy			
332	T15	46,XN	arr[hg19] chr15 × 2 hmz^a^	Termination of pregnancy			
Group: discordant cases							
6	T14	46,XN,−14,+rob(14; 21)	Not available	Termination of pregnancy			
20	T15	47,XXY[21]/46,XY[69]	Not available	37^+6^	Male	2,650	SGA
144	T20	46,XN,inv(20)(p13q13.1)	arr[hg19] (1–22)×2	40^+1^	Male	3,700	Normal
334	T15	45,XN,der(14,15)(q10:q10)[109]/46,XN[8]	arr[hg19] (1–22)×2	38^+6^	Female	2,320	SGA

^a^ This case was confirmed as maternal uniparental disomy of chromosome 15 by methylation analysis.

SGA, small-for-gestational-age; PTB, preterm birth.

### 3.4 Pregnancy outcomes of positive cases

**FIGURE 3:**
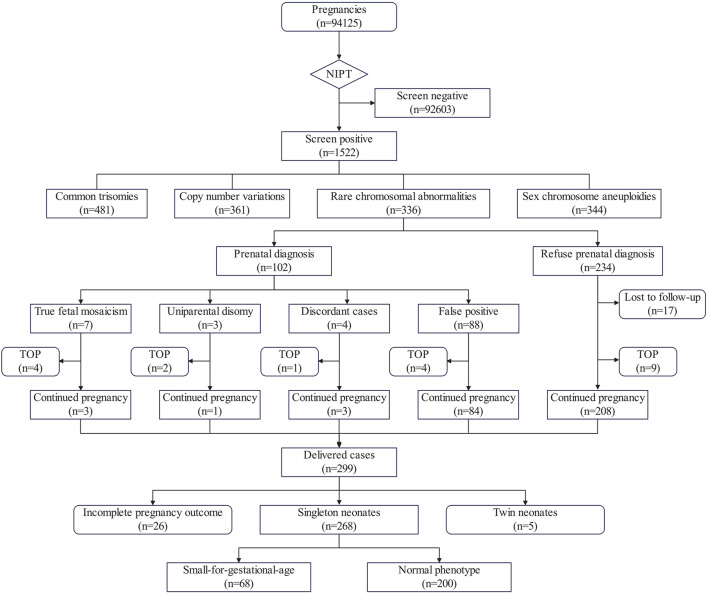
Flowchart of pregnancy outcomes following NIPT positive for RCAs. TOP, termination of pregnancy.

Among 336 RCAs-positive cases, 17 were lost to follow-up and 20 underwent termination of pregnancy for the following reasons: 3 cases due to early fetal demise, 3 cases due to oligohydramnios in mid-late gestation, 3 cases due to fetal developmental anomalies, 2 cases due to placental complications, 2 cases due to maternal anxiety, four true-positive mosaic RATs cases, 1 T21 case and 2 UPD cases confirmed by prenatal diagnosis (Figure 3). Follow-up data were obtained for 299 cases, among which 26 reported only fetal health status without detailed outcomes. Complete pregnancy and fetal development data were ultimately collected for 273 cases (5 twin and 268 singleton pregnancies). Within this cohort, 35 delivered before 37 weeks, resulting in a preterm birth rate of 12.82%. Compared with the preterm birth rate (845/8505, 9.94%) at our hospital in 2024, the difference was not statistically significant (P = 0.118).

**FIGURE 4:**
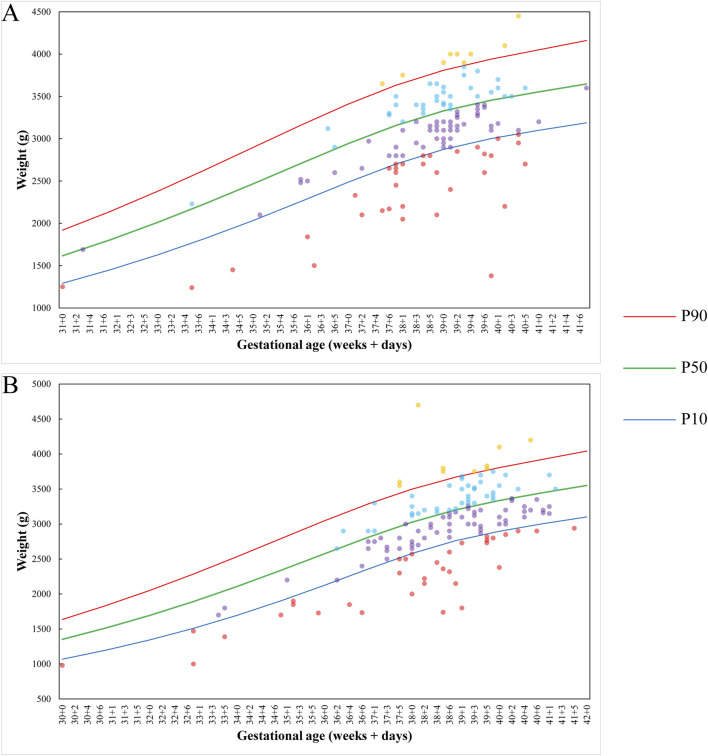
The weight distribution of singletons born with NIPT detection of RCAs. **(A)** Male. **(B)** Female. Red dots represent neonates with birth weight below the 10th percentile; purple dots indicate birth weight between the 10th and 50th percentiles; blue dots correspond to birth weight between the 50th and 90th percentiles; yellow dots signify birth weight above the 90th percentile.

According to the *Growth Assessment Standards for Newborns at Different Gestational Ages* issued by China’s National Health Commission, 68 of 268 singleton neonates (25.37%) were SGA (Figure 4). Subgroup analysis showed no significant sex-based difference: SGA rates were 25.98% (33/127) in males (Figure 4A) versus 24.82% (35/141) in females (P = 0.827) (Figure 4B). Similarly, no statistical difference existed between T7 cases (22.22% SGA, 18/81) and non-T7 RCAs (26.74% SGA, 50/187) (P = 0.435). Remarkably, all three neonates from true-positive mosaic RATs cases that continued to delivery were SGA, and two of them were preterm birth (Table 2).

### 3.5 Placental, peripheral blood, and familial validation

**FIGURE 5:**
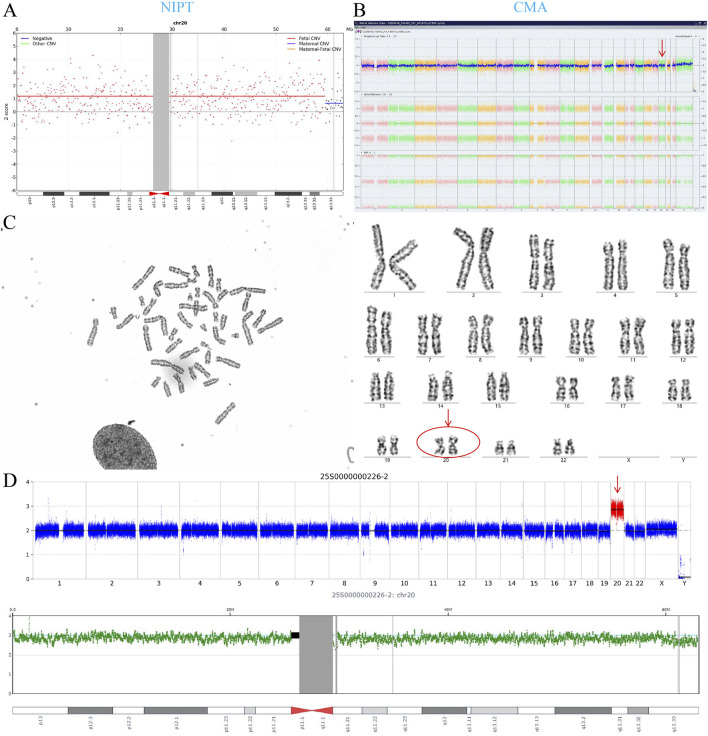
NIPT-Detected trisomy 20 with Negative Amniotic Karyotype and CMA, but Placental trisomy 20 by CNV-seq. **(A)** NIPT revealed trisomy 20. **(B)** Amniotic fluid CMA showed a normal female karyotype (46,XX). **(C)** Amniotic fluid karyotype analysis confirmed euploidy (46,XX) with 47 cells counted. **(D)** CNV-seq of the placenta identified trisomy 20.

**FIGURE 6:**
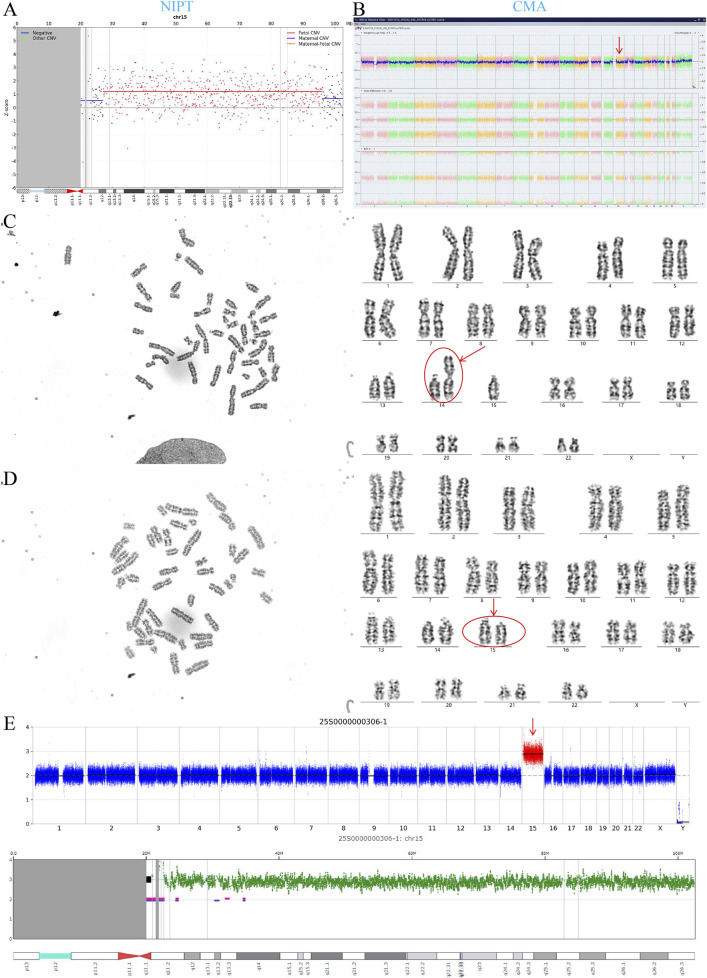
NIPT-Detected trisomy 15 with Discordant Amniotic Findings: Mosaic Robertsonian Translocation by Karyotype but Negative CMA, and Placental trisomy 15 by CNV-seq. **(A)** NIPT revealed trisomy 15. **(B)** Amniotic fluid CMA showed a normal female karyotype (46,XX). **(C)** Amniotic fluid karyotype analysis identified mosaicism for a Robertsonian translocation [45,XX,der(14; 15)(q10; q10)] in 109 of 117 cells (93.2%). **(D)** A normal karyotype (46,XX) was observed in 8 of 117 cells (6.8%), confirming mosaicism. **(E)** CNV-seq of the placenta identified trisomy 15.

CNV-seq analysis was performed on placental tissues from four false-positive cases with NIPT-positive/prenatal diagnosis-negative results. Two cases showed negative CNV-seq results across six sampled placental sites (Supplementary Figures S3, S4). One case (NIPT: trisomy 20; prenatal diagnosis: negative) exhibited placental trisomy 20 (Figure 5), while another (NIPT: T15; prenatal diagnosis: 45,XN,der(14; 15)(q10; q10)[109]/46,XN[8]) demonstrated placental trisomy 15 (Figure 6). Additionally, peripheral blood from a neonate with prenatally confirmed 10% mosaic T16 underwent karyotyping and CNV-seq, both yielding negative results.

In a discordant case where NIPT indicated T14 but prenatal diagnosis revealed 46,XN,−14,+rob(14; 21)(q10; q10), placental CNV-seq confirmed T14. Familial analysis revealed a paternal Robertsonian translocation (45,XY,der(14; 21)(q10; q10)) and a normal maternal karyotype (46,XX).

## 4 Discussion

NIPT technology detects fetal genetic information indirectly by analyzing cffDNA present in maternal peripheral blood (Hanson et al., 2022). Primarily derived from apoptotic placental trophoblast cells, cffDNA constitutes 5%–20% of total cell-free DNA in maternal circulation, and its proportion increases with gestational age (Zaki-Dizaji et al., 2023). Initially, NIPT relied on targeted capture or massively parallel shotgun sequencing to identify specific fetal chromosomal abnormalities, such as common trisomies or selected CNVs (Dungan et al., 2023; Holesova et al., 2024). Recent advancements in sequencing and cffDNA enrichment, particularly in China, now enable low-coverage sequencing of the entire fetal genome via next-generation sequencing and bioinformatics analysis, achieving reliable detection of CNVs as small as 1–2 Mb (Shen et al., 2024; Wang et al., 2024). Given that most RCAs involve chromosomal segments significantly larger than those in common trisomies, NIPT holds theoretical potential for RCAs detection. In this study, we analyzed 94,125 NIPT cases to evaluate its performance in screening RCAs, focusing on sensitivity, specificity, and clinical utility. This investigation, featuring a larger sample size (N = 94,125) and more comprehensive pregnancy outcome data than many previous studies, extends beyond the sole focus on prenatal diagnostic PPV.

Our study revealed a positive rate of 0.36% for RCAs by NIPT, predominantly RATs, with only a minimal proportion attributed to RAMs, likely due to early embryonic lethality associated with RAMs (Gu et al., 2021; Lebedev et al., 2004). The RATs-positive rate in our cohort was slightly higher than the average rate reported in 10 cffDNA studies (634 of 196,662 samples; 0.32%) (Benn et al., 2019). Our findings align with most prior studies (Konya et al., 2024), with T7 representing the most prevalent RCAs. Other frequent RCAs included trisomy 8, trisomy 20, trisomy 3, and trisomy 16, though distribution rankings varied across cohorts (Konya et al., 2024; van Eekhout et al., 2023; Zhang et al., 2023). We hypothesize that these discrepancies may reflect random variability stemming from differences in sample sizes and testing platforms among studies.

**FIGURE 7:**
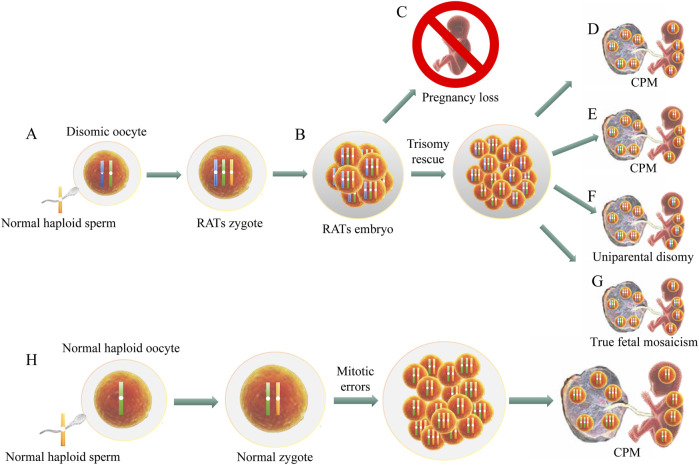
Mechanisms of embryonic mosaicism: trisomic rescue and zygotic mitotic errors. **(A)** Rare Autosomal Trisomies (RATs): Zygote formation via fertilization of a disomic oocyte (maternal meiosis I non-disjunction) by a normal haploid spermatozoon. **(B)** Trisomic rescue mediates chromosomal mosaicism in RATs embryos. **(C)** Most RATs lead to pregnancy loss. **(D,E)** Trisomic rescue via loss of a maternal chromosome induces confined placental mosaicism (CPM). **(F)** Trisomic rescue with paternal chromosome loss generates CPM and fetal uniparental disomy of maternal origin. **(G)** True fetal mosaicism occurs when trisomic rescue fails to eliminate all aneuploid cells, preserving RATs lineages in fetal tissues. **(H)** CPM with placental RATs and euploid fetus originates from trophoblast mitotic errors during early embryogenesis. **(A–G)** Illustrate trisomic rescue in RATs embryos from disomic oocyte fertilization; **(H)** Depicts trophoblast-specific mitotic errors.

RCAs, as a subtype of chromosomal aneuploidy, are generally caused by errors in chromosome segregation during meiosis or mitosis (Klaasen and Kops, 2022). Most chromosomal aneuploidies result from meiotic errors, particularly during oocyte meiosis (Figure 7A), which are strongly correlated with maternal age (Charalambous et al., 2023). Advanced maternal age increases the likelihood of chromosomal aneuploidy, as exemplified by the well-documented positive association between maternal age and common chromosomal aneuploidy such as T21 (Hultén et al., 2010). In our cohort, maternal age was significantly higher in common trisomy and non-T7 RCAs cases than in NIPT-negative and CNV-positive groups (P < 0.05), supporting predominant attribution to age-related meiotic errors in oocytes (Lannoo et al., 2022). Conversely, T7 cases exhibited lower maternal age than common trisomy and non-T7 RCAs (P < 0.05) but comparable to NIPT-negative and CNV-positive groups, indicating maternal age-independent origins likely through postzygotic mitotic errors during embryonic cleavage (Currie et al., 2022). Due to sample size limitations, only T7 was analyzed as a distinct subgroup to explore its potential mechanisms, while other RCAs were collectively assessed, which may cause bias. We will expand sample cohorts to validate our findings in future research.

Our prenatal diagnostic data revealed a PPV of 6.86% for NIPT in detecting RCAs, exceeding the 4.1%–6% range reported in prior literature (Van Den Bogaert et al., 2021; van der Meij et al., 2019; Zhang et al., 2023). Notably, our PPV (6.86%) differs from Liu et al.’s report of 13.51% (Liu et al., 2023). This discrepancy stems from differing inclusion criteria: Liu et al. classified UPD and partial deletions as RCAs true positives, whereas we defined RCAs strictly as complete numerical chromosomal abnormalities. Recalculating their PPV using our definition yields 8.11% (3/37), which aligns with our finding of 6.86% (P = 0.802). We identified three cases with false-negative karyotyping results (30 metaphases analyzed), but CMA results were positive. These results suggested that certain RATs may involve proliferation defects in cultured cells, which may generate an underestimation of abnormal cell proportions—a phenomenon also observed in other studies (C. P. Chen et al., 2011; Chen et al., 2013; Chen et al., 2015). Although karyotyping (typically analyzing 20–30 metaphases) remains the gold standard for detecting chromosomal aneuploidy, culture-induced proliferation bias may distort the true chromosomal composition. Molecular diagnostic methods like CMA and CNV-seq could bypass cell culture and mitigate this bias, but these methods were not routinely adopted due to higher costs. A study explicitly employing CMA, fluorescence *in situ* hybridization and CNV-seq achieved a PPV of 22% (10/46) (Xie et al., 2023). Early prenatal diagnostics relied solely on karyotyping in our laboratory. However, after recognizing false-negative karyotypes, we now recommend concurrent karyotyping and CMA for NIPT-positive RCAs, which may explain our elevated PPV compared to other cohorts. Although 400-band karyotyping adequately detects numerical chromosomal abnormalities, its limited resolution precludes reliable identification of low-level mosaicism and submicroscopic structural anomalies. This constitutes a recognized limitation of the present study. We plan to implement 550-band analysis (resolution 2–5 Mb) in future investigations to improve detection accuracy.

The low PPV of NIPT for RCAs primarily stems from biological and methodological false positives. Biologically (Figure 7C), most RCAs cause early pregnancy loss due to lethal effects (Essers et al., 2023). However, rare cases involve trisomy rescue in RATs (Figure 7B), where embryonic cells eliminate an extra chromosome to restore euploidy (Akutsu et al., 2022). When placental cells retain the trisomy while embryonic cells become euploid (Figures 7D,E), confined placental mosaicism occurs (Baptiste et al., 2025)—the predominant scenario in our cohort. If trisomy rescue eliminates a parental chromosome during embryogenesis (Figure 7F), this process is termed UPD (Benn, 2021). True fetal mosaicism (Figure 7G) is diagnostically defined by coexisting rescued euploid and residual RATs cell populations within embryonic tissues (Basaran et al., 2022). Mitotic errors (Figure 7H), frequent in rapidly dividing trophoblasts, can result in placental-specific RATs (e.g., T7, T8) alongside normal fetal cells (Lannoo et al., 2022). This biological discordance explains both the high NIPT detection rate and low PPV observed for T7. Thus, biological discordance from trisomy rescue or mitotic errors is an unavoidable cause of low PPV. Methodological false positives, driven by GC-bias, data noise, algorithmic limitations or microdeletions/duplications altering Z-scores, also contribute to low PPV (Benn and Cuckle, 2023). Unlike biological false positives, these yield negative results upon placental tissue analysis, as observed in two cases here. Future technical refinements such as algorithmic optimization, data noise reduction, and interpretation protocol enhancement will mitigate methodological false positives, thereby improving the efficacy of NIPT for detecting RCAs.

However, the low PPV does not diminish the clinical significance of NIPT-detected RCAs, as numerous studies associate RCAs-positive cases with elevated risks of adverse pregnancy outcomes (M. Chen et al., 2025; Yan et al., 2025; Zhang et al., 2023). In our cohort, 10 RCAs-positive cases without prenatal diagnosis underwent termination due to fetal, amniotic, or placental complications. These cases indicate that fetal loss represents a potential consequence of RCAs positivity. Although preterm birth is frequently cited as an adverse outcome (Chen et al., 2025; Yan et al., 2025), our observed preterm rate aligned with the regional baseline (P = 0.118), suggesting no direct attribution to RCAs. In our cohort study, gestational age at delivery and birth weight were recorded using direct measurements rather than ultrasound estimates, revealing that 24.81% of singleton neonates were SGA. This rate substantially exceeded the regional baseline (8.92%), indicating that SGA represents a clinically significant adverse pregnancy outcome in RCAs-positive pregnancies. Although T7 and other RCAs arise from distinct pathogenic mechanisms, their SGA rates showed no statistically significant difference (P = 0.435). This suggests that placental mosaicism may contribute to fetal growth restriction irrespective of its mechanistic origin (Del Gobbo et al., 2021; Spinillo et al., 2022). Critically, all three true fetal mosaicism cases exhibited SGA, supporting fetal chromosomal mosaicism as an etiological factor. Current methodologies cannot distinguish whether SGA in these cases originates from placental RCAs (confined placental mosaicism) or true fetal mosaicism. This etiological distinction warrants targeted investigation.

Additionally, UPD detected prenatally adversely impacts pregnancy outcomes by elevating risks of imprinting disorders and recessive genetic diseases (Elbracht et al., 2020; Papenhausen et al., 2021). Maternal UPD involving chromosomes 7, 11, 14, 15, and 20 can cause specific phenotypes, while paternal UPD of chromosomes 6, 11, 14, 15, and 20 is associated with distinct clinical manifestations (Del Gaudio et al., 2020). Therefore, the prenatal detection of UPD, even as an explanation for a discordant NIPT RCAs result, carries significant clinical implications requiring specialized genetic counseling and potential postnatal follow-up.

## 5 Conclusion

In this cohort of 94,125 pregnancies, we found that most RCAs were associated with maternal age and meiotic errors, while T7 arose independently of maternal age, likely through mitotic errors. Concurrent use of karyotyping and CMA, rather than karyotyping alone, reduces culture bias and improves PPV. Both biological and methodological factors contribute to the low PPV of NIPT for RCAs. Despite suboptimal PPV, RCAs-positive cases exhibit significantly elevated risks of fetal loss, SGA, and UPD, though not preterm birth. Thus, NIPT-detected RCAs retain clinical significance for risk stratification and pregnancy management.

## Data Availability

The original contributions presented in this study were publicly available. This data can be found in Figshare https://doi.org/10.6084/m9.figshare.29266616.

## Supplementary material

SUPPLEMENTARY TABLE S1 Summary of 336 positive NIPT results with RCAs.

SUPPLEMENTARY FIGURE S1 Case with negative initial standard karyotype but T16 mosaicism identified by CMA and extended-count karyotype analysis.

SUPPLEMENTARY FIGURE S2 Case with negative initial standard karyotype, T16 mosaicism identified by CMA, and negative extended-count karyotype analysis (130 Cells).

SUPPLEMENTARY FIGURE S3 Case with two consecutive NIPT results of trisomy 20 but negative prenatal karyotype, CMA, and CNV-seq (6 placental loci).

SUPPLEMENTARY FIGURE S4 Case with NIPT-detected T3 but negative prenatal karyotype, CMA, and CNV-seq (6 placental loci).
