# Supplementary figures and images for: Performance and clinical implications of non-invasive prenatal testing for rare chromosomal abnormalities: a retrospective study of 94,125 cases
Source: Front Mol Biosci. 2025 Aug 20;12:1645223. doi: 10.3389/fmolb.2025.1645223 (PMC12404953; doi:10.3389/fmolb.2025.1645223)

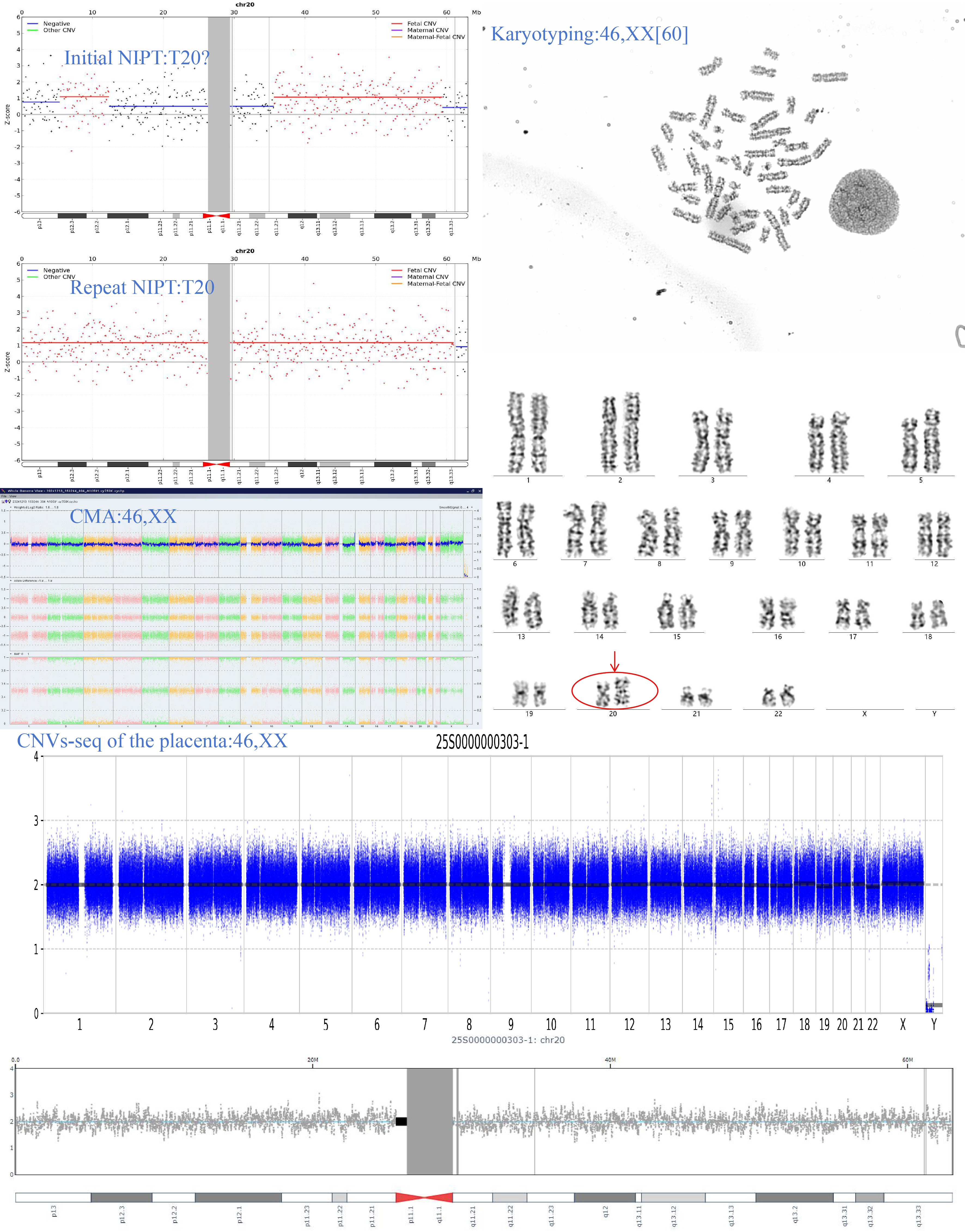

Supplement: Supplementary file 1 [file Image3.jpeg]

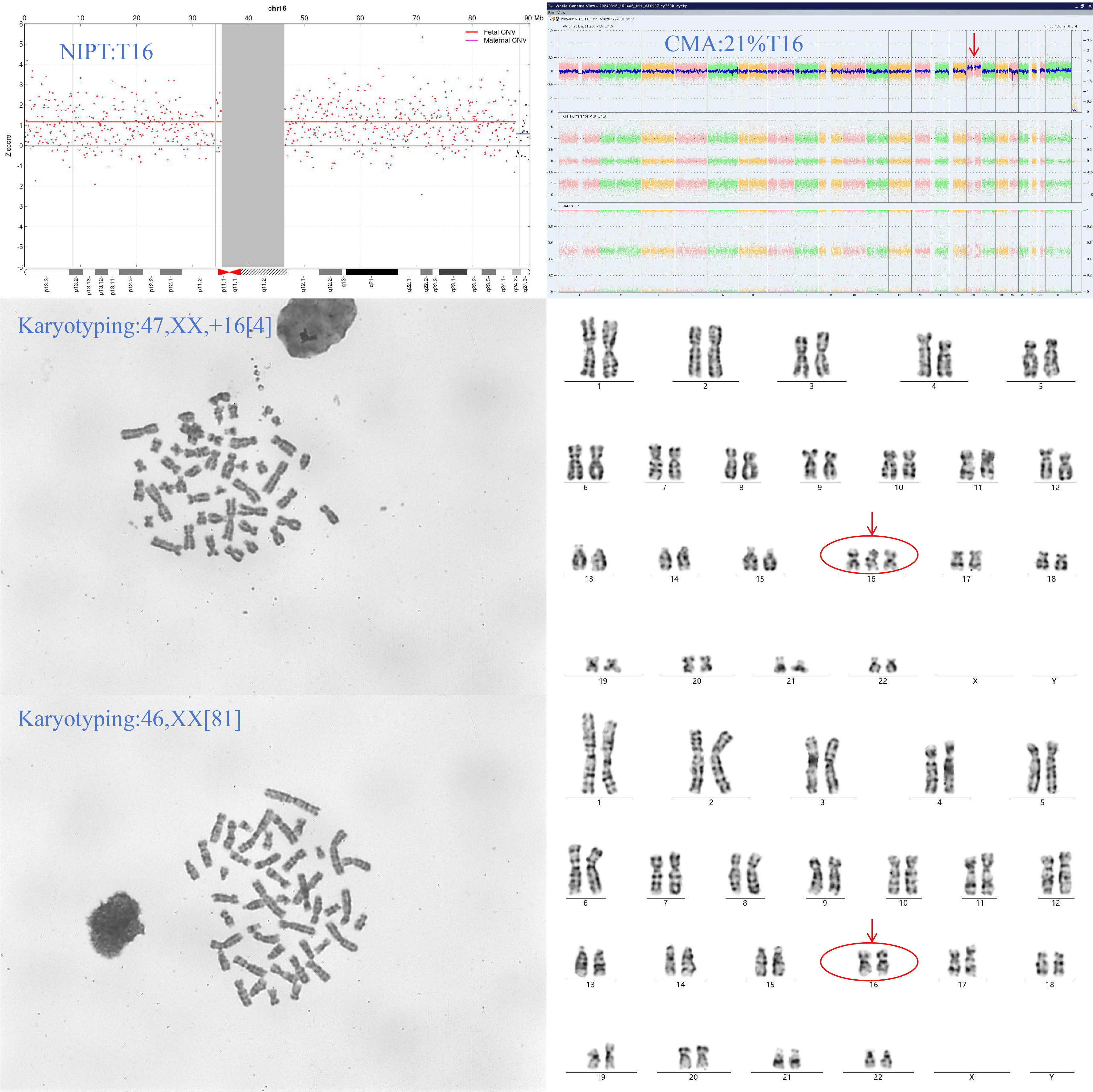

Supplement: Supplementary file 2 [file Image1.jpeg]

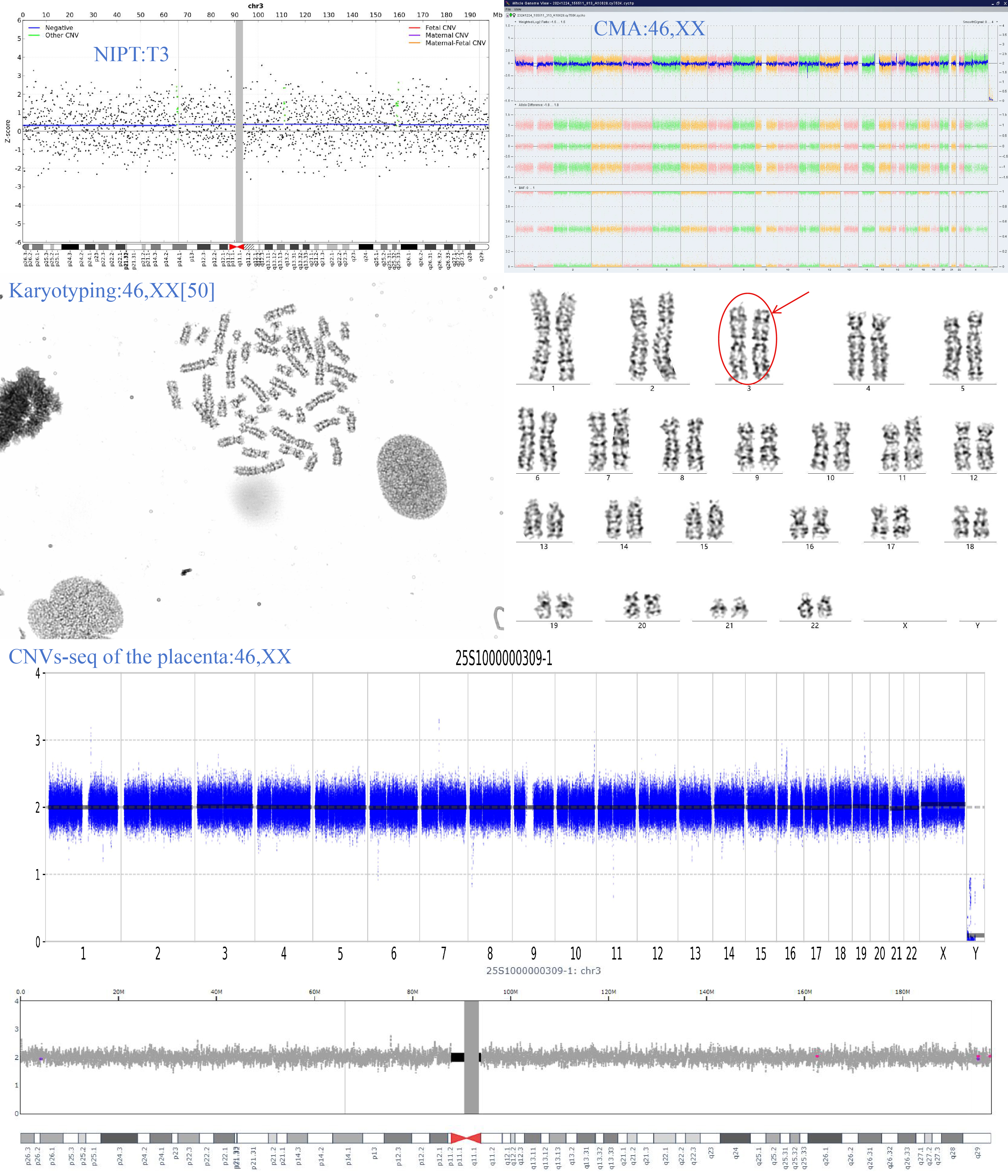

Supplement: Supplementary file 3 [file Image4.jpeg]

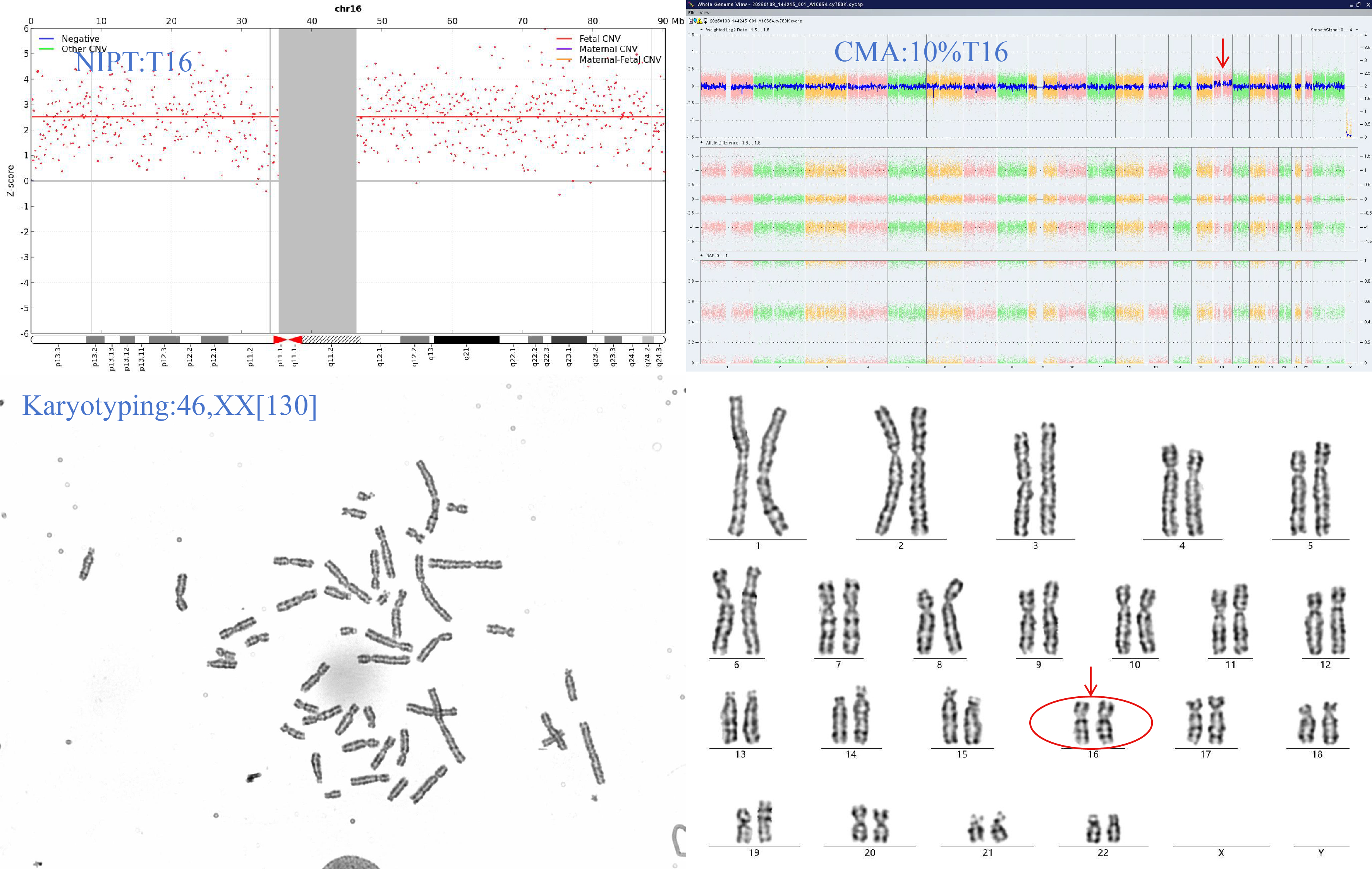

Supplement: Supplementary file 4 [file Image2.jpeg]
